# Wireless local area network in a prehospital environment

**DOI:** 10.1186/1472-6947-4-12

**Published:** 2004-08-31

**Authors:** Dongquan Chen, Seng-jaw Soong, Gary J Grimes, Helmuth F Orthner

**Affiliations:** 1 Health Informatics Program, Department of Health Services Administration School of Health Related Professions. University of Alabama at Birmingham (UAB). Birmingham, Alabama, USA; 2 Current Address: Biostatistics and Bioinformatics Unit, Comprehensive Cancer Center of UAB. Birmingham, Alabama, USA; 3 Department of Electrical & Computer Engineering, Centre for Telecommunications of UAB. Birmingham, Alabama, USA

## Abstract

**Background:**

Wireless local area networks (WLANs) are considered the next generation of clinical data network. They open the possibility for capturing clinical data in a prehospital setting (e.g., a patient's home) using various devices, such as personal digital assistants, laptops, digital electrocardiogram (EKG) machines, and even cellular phones, and transmitting the captured data to a physician or hospital. The transmission rate is crucial to the applicability of the technology in the prehospital setting.

**Methods:**

We created two separate WLANs to simulate a virtual local are network environment such as in a patient's home or an emergency room (ER). The effects of different methods of data transmission, number of clients, and roaming among different access points on the file transfer rate were determined.

**Results:**

The present results suggest that it is feasible to transfer small files such as patient demographics and EKG data from the patient's home to the ER at a reasonable speed. Encryption, user control, and access control were implemented and results discussed.

**Conclusions:**

Implementing a WLAN in a centrally managed and multiple-layer-controlled access control server is the key to ensuring its security and accessibility. Future studies should focus on product capacity, speed, compatibility, interoperability, and security management.

## Background

The development of the Internet has encouraged doctors to use computers and hospitals to use wireless communications [1], since wireless technology offers many benefits over its wired counterpart, including ease of installation and access to network information [2-6], and higher productivity and convenience [2]. One study using personal digital assistants (PDAs) connected to a network showed that the device was of limited use in transmitting data in prehospital stroke management [7]. Another study showed that cellular phones, pagers, or other radio-based devices will remain an important communication mode in the near future [8]. The advancement of wireless local area network (WLAN) technology provides the potential to allow physicians to obtain a patient's information anywhere, even before the patient reaches the emergency room (ER) (Orthner, personal communication). Timely access to a patient's information may fundamentally improve patient care [9] in both pre- and in-hospital settings, due to earlier doctor interventions.

At present, patient data such as electrocardiograms (EKG) and demographics are seldom sent from the prehospital environment to the ER before the ambulance arrives [10]. As a result, some preventive measures have to be given, regardless of need (e.g., aspirin [11] or thrombolysis [12] for presumed acute myocardial infarction. However, despite the potential benefits of wireless technology in prehospital settings, the application of this technology has been slow and few related studies have been carried out.

The objective of this study was to assess the ability of wireless technology to facilitate data communication between a prehospital setting and an ER (Orthner, personal communication). The idea was for all the data collected by paramedical personnel to be transmitted to an ER server from the patient's home, on the way to the ER, or upon arrival at the ER. Thus the transmission rate is crucial to the usefulness and applicability of the technology. To test the feasibility of wireless data transmission under the various scenarios, two separate WLANs were created, one around our office and another in a house. In this report, we discuss our testing of the wireless technology, and its potentials and limitations in simulated prehospital settings.

## Methods

The WLAN products used (Aironet 340 and 350 series wireless client adaptors and access points (APs); Cisco) offered 11-megabits-per-second (Mbps) transmission rates, built-in security features (including 40- to 128-bit encryption) and Web-based management. The transmission rates of files of different sizes were measured with different APs, patch antennae, clients, and transfer methods. The security of patient data was ensured using a centrally managed Access Control Server (ACS). Other issues such as standards, roaming, and cost are also discussed here.

Within a WLAN, data are transmitted between a server and its wireless clients via an AP antenna. Both workstations and laptops were used here as servers, and file transfer rates were measured for both systems. We used Gateway Select series, Dell Inspiron series, and a Toshiba Satellite laptop computer as clients. The computers had CPU operating at 0.8–1.2 GHz, 256–1024 MB of RAM, and 10–40 GB hard drives, and all ran the Microsoft Windows 2000 Professional operating system. PDAs (Ipaq Pocket PCs, models 3550 and 3570, 200-MHz CPU, 32–64 MB RAM, Compaq) were also tested as wireless clients. The wireless coverage was tested using two APs (Aironet 340 and 350 series, Cisco) and a patch antenna (S2406P, Cushcraft Corporation). The feasibility of using cellular phones (StarTac 7868, Motorola) in data transmission in the area not covered by the WLAN was also tested.

The various software used in the study for wireless client management, file transfer, and access control included the Aironet Client Utility (Cisco), Link Status Meter (LSM, Cisco), the ACS (V3.0, Cisco), Phone Tools (BVRP Software) for faxing, and file transfer protocol (FTP) for measuring the file transmission rate. The software LSM classifies the link status as the percentage of maximum signal strength and quality: "excellent" (>75%), "good" (40–75%), "fair" (20–40%), or "poor" (<20%); where signal strength and quality refer to the client adapter's radio signal at the time packets are being received, quantified as bytes transmitted and received and the errors that occur. Detailed descriptions of the mentioned software are available from the manuals provided by the vendors.

### The WLAN and its configuration

The Aironet 340 and 350 series APs were tested by a two-step approach. In the first step, one AP was connected directly with the server that was not connected to the campus Ethernet backbone. In the second step, the AP was assigned a public Internet Protocol (IP) address and connected to the Ethernet backbone in the Susan Mott Webb Nutrition (Webb) Building at the University of Alabama at Birmingham. The IP address was assigned to an AP through either HyperTerminal or a Web console using a Web browser. An administrator ID and password were then created to enhance the Web console security. The client computer required a type II PCMCIA (Personal Computer Memory Card International Association) card slot. Every client needed a functional IP address to become associated with the AP. The Wired Equivalent Privacy (WEP) keys were enabled for both the AP and the clients to ensure two-way authentication.

### Comparison of different coverage of APs and patch antennas

The coverage of the WLAN was found to highly structure dependent. The floor of the Webb building measures about 60 by 25 meters. A single Aironet 340 AP was unable to cover the entire floor with a "good" link status. This was achieved using two (more powerful) Aironet 350 APs. Achieving the "excellent" link status on the floor required the use of the S2406P (Cushcraft) patch antenna. The wireless clients associated with the AP had a "fair" or "good" status one floor up and one floor down from the floor where the AP was located. There was a small area outside the 5-floor building in which the clients could associate with the AP with a "poor" status.

## Results

### Link statuses around a simulated patient's home

**Figure 1:**
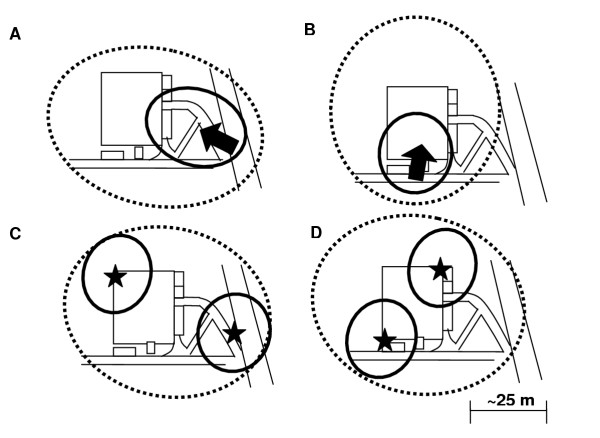
**An aerial view of the use of a patch antenna at a simulated patient's home. **The AP – clients connection (A and B) and peer-to-peer connection (C and D) configurations were tested. The arrows indicate the locations and the beaming directions of the APs with a patch antenna. The stars indicate the locations of the wireless devices in the peer-to-peer connections. The link statuses were measured as described in the Methods: the solid and dashed circles indicate "excellent" and "good" statuses, respectively. Panel A: Simulated ambulance parked on the street. Panel B: Simulated ambulance parked in the parking lot of the house. C and D: Peer-to-peer connection was created between two wireless clients.

In order to test the feasibility of implementing an isolated LAN around a patient's home, we chose a two-story house with the layout shown in Figure 1. Two simulated scenarios were tested: one with an ambulance parked on the street next to the house (Figure 1A and 1C), and the other with the ambulance parked next to the house in its parking lot (Figure 1B and 1D). Two connection modes under each scenario were also tested: one mode used the AP-client connection (Figure 1A and 1B) and the other used a peer-to-peer connection without the AP (Figure 1C and 1D). The link statuses both inside and outside the house were at least "good", and some of the area close to the AP in both scenarios had "excellent" coverage. This suggested that an ambulance with a patch antenna could communicate at a reasonable rate with handheld devices operating inside the patient's home, through either an AP or a direct peer-to-peer connection.

### File transfer rate with laptops

To quantify the baseline signal strength, one Aironet 350 AP was tested in the open area: "excellent", "good", "fair", and "poor" link statuses were obtained within 10, 25, 50, and 100 feet (~4, 10, 20, and 39 meters), respectively. As shown in Table 1A, the file transfer rate for a 10-MB file was 224–2,000 kbps, depending on the distance. Interestingly, higher rates were reached for files of size 10–100 MB.

The different methods and directions of file transfer might affect the rate. Methods such as FTP transfer, copying and pasting between folders, and Microsoft Briefcase synchronization were tested. Other factors that may affect transmission were also tested, including the initiation direction (pulling or pushing, in terms of the choice of client and server; see below) and relative physical motion. One Gateway workstation and two Inspiron 4000 laptops were used. To simplify the test, a single medium-sized file (50 MB) was chosen for the purpose. We chose a 50-MB file since it corresponds to a typical high-quality EKG image. As indicated in Table 1B, pushing refers to a transfer from the server to clients if initiated from the server side, and from a client to the server if initiated from the client side; whereas pulling refers to the transfer from the server to clients if initiated from the client side, and from a client to the server if initiated from the server side. Pulling a file led to a higher rate of transfer, in all link statuses (i.e., distances). The speed was lower when two clients transferred the 50-MB file at the same time, and it was generally lower for file transfer between two clients than between a server and a client (Table 1B, Sections III-IV). As expected, the transfer rate was slower while the client device was moving (as shown in Table 2B, section V). However, it was still found that in a WLAN environment, paramedics carrying data-collecting devices could move around and transmit data by different methods and directions at a reasonable rate. The simultaneous file transfer that involves multiple clients/users is a more likely scenario in real emergency settings. Under a "good" link status, the ability of five laptop computers to pull a single file from the FTP server was tested both individually (Table 2A) and simultaneously (Table 2B). The data rate reached 5.9 Mbps when a single client was transferring, and fell to below 2 Mbps when multiple clients were involved simultaneously (based on four independent tests). This lower rate, however, is still within a reasonable range, considering the file size.

### File transfer rate with Pocket PC and cellular phone

We chose Ipaq Pocket PCs as PDAs due to their relatively large amount of RAM compared to other PDAs, and a cellular phone (Motorola) as alternative data transmission device when the PDA was outside the WLAN coverage (to simulate the scenario when a long-range antenna, such as a yagi antenna (Cisco), is not available). The transfer rates for single files of different sizes are summarized in Table 3A. The faxing speed through the phone did not appear to be correlated with the file size, since a 50-fold difference in file size resulted in a 30% difference in the time needed to complete the file transmission. The reason is unknown, and should be further investigated.

### Enhancing the WLAN security using an ACS

**Figure 2:**
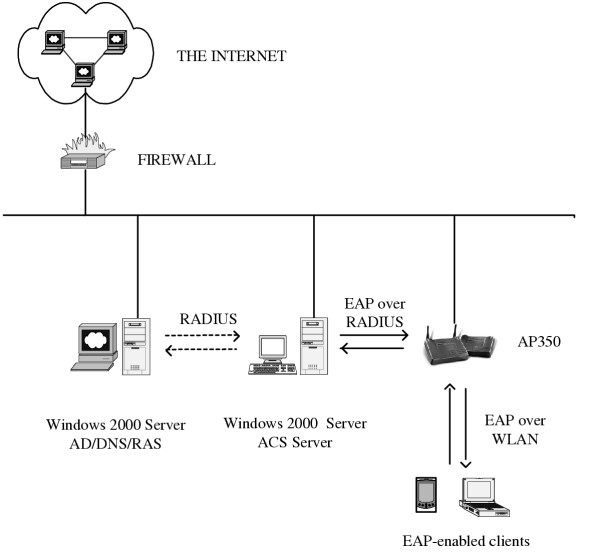
**The Cisco Secure ACS and its configuration. **The AP was configured to use ACS AAA services for the Extensible Authentication Protocol (EAP)-enabled wireless devices over the WLAN. A Windows 2000 Server running Active Directory (AD) was used to mimic a network access server (NAS) to negotiate with ACS through the Remote Access Dial-in User Service (RADIUS) protocol. The NAS had an enabled RAS connection for the ACS.

A Cisco V3 ACS was used to improve the security of the network [13]. As shown in Figure 2, the Active Directory facility of a Windows 2000 Server was used as a network access server to communicate with the ACS for authentication, authorization, and accounting (AAA) [14]. The ACS was able to control client access to the network through the AAA process.

### A private local area network

**Figure 3:**
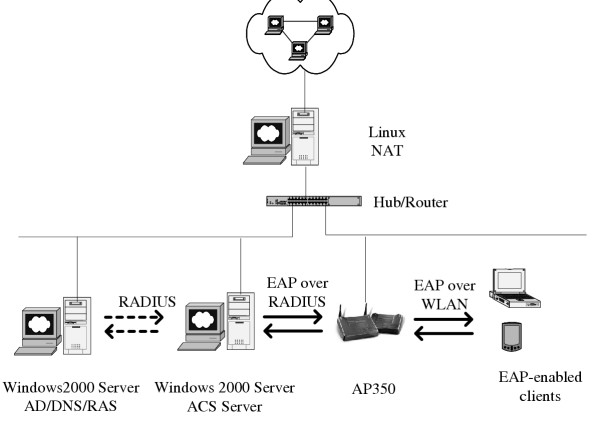
**A private WLAN with an ACS. **A workstation running the Linux operating system functioned as a Network Address Translation (NAT) server. The private LAN was linked with the Internet through a switched hub or router. The AP was configured to use ACS for AAA services for the EAP-enabled wireless devices over the WLAN. A Windows 2000 Server running Active Directory was used to mimic a NAS to negotiate with ACS through the RADIUS protocol.

When managing a WLAN with many clients, there are often insufficient public IP addresses. The solution is to use either the Dynamic Host Configuration Protocol (DHCP) or a private LAN with a Network Address Translation (NAT) server. DHCP services are not easily managed and sometimes create security challenges to network administrators in determining the user's identity. A private LAN seems to be a better solution and more applicable in most ER environments where many wireless clients may transmit at same times, thus maintaining relatively high-speed connections. Figure 3 shows a private LAN with a NAT server that was configured and tested. All wireless clients were centrally managed through an ACS server. The file transfer rates were similar to those achieved with public IP addresses under similar conditions (data not shown).

### Cost of a small-scale WLAN

Health-care organizations have traditionally been slow in accepting WLAN technology in clinical practice. One of the major concerns has been its cost [15,16], followed by security [17-20], although the benefits of WLANs have been demonstrated in many fields including telemedicine [21]. The cost of a simple WLAN like the one tested here was calculated (Table 4).

## Discussion

### Synchronization

After collecting all patient data at a patient's home, the data must ultimately be transferred to the ER. This involves two critical synchronization steps: (1) from the patient's home to a server on the ambulance, and (2) from the ambulance to the ER (while in transit or upon arrival). Here the WLAN was employed for both of these steps, using Microsoft Briefcase and Windows Workgroups. Automatic synchronization with the destination server and batch synchronization were desired. The ultimate objective is, however, to link the two synchronization steps using a long-range antenna that reaches up to 25 miles (e.g., a yagi antenna from Cisco). This would significantly shorten the time needed to transfer data from a patient's home to the ER, since the data will reach a ER WLAN earlier. We are currently performing the associated experiments. We also tested the use of a cellular phone and other types of PDA (e.g., a Palm Pilot) with network capabilities in transmitting a small (up to 50 MB) but critical file. The results suggested that cellular phones or PDAs with network cards can be effective alternatives to the use of a long-range antenna to transmit data from a patient's home to the ER.

### Wireless transfer of EKG data

EKG data are considered very valuable in the early detection, early intervention, and possibly better outcome of heart attack patients [9]. The use of a wearable device with sensors to monitor specific physiological signals and communicate with a personal server has been reported [22]. Land-based telephone lines have also been employed to transmit EKG data and for monitoring by clinical personnel [23]. In our study, we showed that files up to 5–10 MB (the size of a typical high-quality digitalized EKG image) could be transferred using FTP or other file transfer methods within minutes. Handheld devices such as a Pocket PC and cellular phone may be useful in transmitting EKG files when the ambulance is still at the patient's home, as shown in Table 3. The time required to transmit a file is proportional to its size in the case of a Pocket-PC-to-laptop transfer, but this was found not to be the case between a cellular phone and a fax machine. The reason for this discrepancy is unknown, and needs to be further investigated. The use of a long-range antenna may ultimately be needed to increase the transmission capacity and speed.

### Interferences

The WLAN operates at 2.42 GHz with an output power of 100 mW, which may pose a risk of interference with medical devices using similar frequencies. Previous studies have shown that a WLAN may interfere with medical devices in close proximity [24] but is unlikely to be interfered with by such devices [25]. Further studies are needed to clearly address the possibility. In another study, infrared modems exhibited a similar performance to a wired system even in an electrically noisy environment [26], indicating that infrared wireless connectivity can be safely and effectively used in operating rooms. These studies suggest that a WLAN can be acceptable for use in prehospital settings if careful interference testing is conducted.

### Security and privacy

The major concern over a WLAN is its security [17-20], especially when personal information is involved. It has been reported that the open-air clear-text transmission of WEP keys and MAC addresses increases network vulnerability [13,27-29]. One approach to minimizing the risk is to control the access of remote and/or wireless clients through the Remote Access Dial-in User Service and AP management using the Extensible Authentication Protocol. The regulation by the Healthcare Insurance Portability and Accountability Act may further delay an organization's decision to adopt WLAN technology, although both the Institute of Electrical and Electronic Engineering (IEEE) 802.3 and the OpenAir standard specifications offer security protection (these are the two major standards in the unlicensed commercial 2.4-GHz WLAN market). According to our experiences, the following steps are required to implement a secure WLAN. First, anonymous access should be disabled and the Service Set Identifier of an AP and data encryption key (WEP key) should be enabled. Secondly, a Web console should be used to designate an administrator and manage APs and clients. Thirdly, an ACS server such as Cisco Secure ACS should be implemented to work with Active Directory in order to offer both device- and user-dependent AAA services. Digital certificates should be applied whenever possible for mutual authentication to protect sensitive information through secure server access and secure Web access. In addition, the physical security of the APs, client, and server computers can never be overemphasized.

### Standards and interoperability

The IEEE 802.11 specification addresses both the physical and MAC layers (Orthner, personal communication), and the OpenAir 2.4 interface standard is derived from the Wireless LAN Interoperability Forum [29] and needs to be interoperable with the IEEE 802.11 standards. The 5-GHz band WLAN standard (IEEE 802.11a) will become more popular once its cost decreases and the required components become more widely available. The use of standardized compliant devices facilitates communication and interoperability.

### Limitations of the study

The present study was mainly based on the Windows operating system and Cisco wireless products. IEEE 802.11a products for the next generation of WLANs are emerging quickly from various vendors. Hence the stability, compatibility, and interoperability with other vendors require further evaluation. Although currently it is relatively expensive to implement a WLAN using this new protocol, the prices and capabilities are expected to improve within the near future.

## Conclusions

Application of WLAN technology will help both paramedics and other health-care professionals in their daily acquisition of information in a localized area such as within a patient's home, an office, a small clinic, or an ER. Implementing a WLAN in a centrally managed and multiple-layer-controlled ACS is the key to ensuring its security and accessibility. Future studies should focus on product capacity, speed, compatibility, interoperability, and security management.

## Competing interests

None declared.

## Authors' contributions

D. Chen, the principal investigator, was most involved in conducting the experiments. H.F. Orthner participated in data collection in the simulated patient's home. H.F. Orthner was the sponsor and S.-J. Soong and G.J. Grimes were the advisors of the fellowship awarded to D. Chen from the National Library of Medicine, National Institute of Health, and they contributed significantly to the design, coordination, and performing of experiments.

## Pre-publication history

The pre-publication history for this paper can be accessed here:



## Figures and Tables

**Table 1:** File transfer directions and rates (kbps, mean ± SD, *n *= 4)

**A. File transfer rate in an open area between two laptops for different file sizes and link statuses**				
	***1 MB***	***10 MB***	***100 MB***	***1000 MB***
Excellent	400	2000	1660	980
Good	210	890	1110	830
Fair	90	660	740	660
Poor	60	220	250	340
**B. Transfer rates for a 50-MB file between a server and clients under different link statuses**				
		
**I. Server to one client**				
		
	**Pushing**	**Pulling**		
		
Excellent	3090 ± 70	5540 ± 180		
Good	2870 ± 280	5530 ± 130		
Fair	630 ± 500	2070 ± 550		
**II. One client to server**				
		
	**Pushing**	**Pulling**		
		
Excellent	2600 ± 110	5210 ± 410		
Good	2710 ± 330	4350 ± 1030		
Fair	340 ± 80	750 ± 440		
**III. Two clients to server**				
		
	**Client 1**	**Client 2**		
		
Excellent	2160 ± 60	3130 ± 320		
Good	2140 ± 370	3230 ± 40		
**IV. Client 1 to client 2**				
		
Excellent	2400 ± 70			
Good	2560 ± 130			
**V. Server to one client while moving**				
		
Excellent	2500 ± 500			
Good	1200 ± 410			

**Table 2:** Rate of file transfers involving multiple clients (kbps, mean ± SD, *n *= 4)

**A. Transferring files individually**			
***Client***	***50 MB***		

Gateway Solo	5830 ± 1340		
Toshiba Satellite 350	5250 ± 600		
Inspiron 8000	5260 ± 990		
Inspiron 4000-1	4890 ± 490		
Inspiron 4000-2	5960 ± 910		
**B. Transferring files simultaneously**			

***Client***	***50 MB***	***40 MB***	***30 MB***

Gateway Solo	1330 ± 180	1430 ± 140	1740 ± 490
Toshiba Satellite 350	1600 ± 340	1580 ± 160	1610 ± 240
Inspiron 8000	1400 ± 190	1470 ± 140	1500 ± 130
Inspiron 4000-1	1490 ± 200	1580 ± 240	1590 ± 260
Inspiron 4000-2	1240 ± 440	1540 ± 190	1560 ± 280
	
	***20 MB***	***10 MB***	***1 MB***
	
Gateway Solo	1690 ± 200	1900 ± 400	7140 ± 110
Toshiba Satellite 350	1850 ± 60	1860 ± 350	6830 ± 120
Inspiron 8000	1730 ± 170	1820 ± 220	4360 ± 2800
Inspiron 4000-1	1690 ± 190	2000 ± 470	3780 ± 1060
Inspiron 4000-2	1840 ± 310	1770 ± 400	3160 ± 1400

**Table 3:** Pocket PC and cellular phone file transfers (SD: 10~25%, *n *= 4)

**A. Pocket PC to laptop**		**B. Cellular phone to a fax machine**	
***File size (kB)***	***Seconds***	***File size (kB)***	***Seconds***
10	<1	10	134
100	2	100	155
1000	15	500	166
5000	150	1500	180

**Table 4:** Cost of a WLAN of similar scale to the one implemented here

***Component***	***No. needed***	***Unit price (US $)***	***Total US$***
Workstation and accessories	1	2500	2500
Laptop and accessories	2	2500	5000
Pocket PC and accessories	3	600	1800
Palm Pilot and accessories	2	500	1000
APs	2	400	800
Patch antennas	2	200	400
Wireless adaptors	5	300	1500
Cellular phone and network costs	1	250	250
		**Total (US $)**	**13250**

**Note**: software costs are usually associated with the purchase of hardware and not included.
